# motA-mediated flagellar motility modulates biofilm formation and competitive nodulation in *Mesorhizobium ciceri* USDA 3378

**DOI:** 10.3389/fmicb.2025.1743961

**Published:** 2026-01-21

**Authors:** Keyu Chen, Haoran Hao, Kaiwen Zhang, Ke Li, Youguo Li, Mitchell Andrews, Hua Zhang, Zhiqiang Feng, Junjie Zhang

**Affiliations:** 1 College of Food and Bioengineering, Zhengzhou University of Light Industry, Zhengzhou, Henan Province, China; 2 College of Life Science and Technology, Huazhong Agricultural University, Wuhan, Hubei Province, China; 3 Faculty of Agriculture and Life Sciences, Lincoln University, Lincoln, New Zealand; 4 Sanquan Food Co., Ltd., Zhengzhou, Henan Province, China; 5 Fujian Chunlun Group Co., Ltd., Fuzhou, Fujian Province, China; 6 Fujian Wenjiechun Agricultural S&T Co., Ltd., Fuzhou, Fujian Province, China

**Keywords:** competitive nodulation, *Mesorhizobium ciceri*, *Mesorhizobium muleiense*, MotA gene, transcriptome

## Abstract

The introduced rhizobial inoculum *M. ciceri* USDA 3378 demonstrates a significant competitive advantage over the indigenous *M. muleiense* CCBAU 83963 for nodulating chickpea in newly established planting areas in China. Previous genomic analyses revealed that USDA 3378 possesses a greater number of genes related to cell movement and flagella production compared to CCBAU 83963. Transcriptomic analysis indicated that the expression of the flagella-associated gene *motA* (flagellar motor protein) significantly changed under symbiotic conditions. Although the genome of *M. ciceri* USDA 3378 contains the *motA* gene, its biological function within this strain has not been previously reported. In this study, we constructed a *motA* mutant (Δ*motA*-3378) in USDA 3378 using homologous recombination and biparental conjugation methods to assess the differences in bacterial structure, growth, motility, exopolysaccharide synthesis, biofilm formation, and competitive nodulation ability between the wild type and the mutant. Experimental results showed that the Δ*motA*-3378 mutant was unable to produce flagella, leading to reduced motility, diminished biofilm formation, and lower exopolysaccharide production. In competitive nodulation with wild-type USDA 3378, the Δ*motA*-3378 mutant's nodule occupancy was 40.43 %. Furthermore, its competitive nodulation advantage against CCBAU 83963 decreased from 100 % (achieved by wild-type USDA 3378) to 94.6 %. These findings indicate that the *motA* gene plays a crucial role in the motility, exopolysaccharide synthesis, biofilm formation, and competitive nodulation ability of *M. ciceri* USDA 3378.

## Introduction

Chickpea (*Cicer arietinum* L.) belongs to the tribe Cicereae, subfamily Papilionaceae of family Fabaceae (Singh and Diwakar, 1995). It is the second most widely grown legume crop after soybean (*Glycine max*) (Bose et al., 2024). According to statistics, chickpea is commercially cultivated in more than 50 countries across Asia, North Africa, Southern Europe, North America, and Australia (Zhang et al., 2024). Chickpea is a temperate legume (Sprent, 2009) with high agronomic value, since it is used for both food and forage (Jodha et al., 1987). Chickpea is used as a good source of proteins and energy in the diets of various organisms including humans and animals (Mukherjee et al., 2024).

Like other legume crops, chickpea can fix atmospheric nitrogen through symbiotic bacteria (rhizobia) in their root nodules, contributing to the production of high content and quality protein in chickpeas (Zhang et al., 2018). Rhizobia are renowned for their ability to induce nodule formation on the roots of leguminous plants, a process that enables the host plant to achieve nitrogen self-sufficiency (Fujishige et al., 2006). At present, the diversity of rhizobia in chickpea has been studied extensively, in which *Mesorhizobium ciceri* and *Mesorhizobium mediterraneum* are the dominant populations in western and South Asian countries, while *Mesorhizobium muleiense* and *Mesorhizobium wenxiniae* are the dominant populations in Northwest China (Andrews et al., 2018; Zhang et al., 2018). In previous studies, we compared the competitive advantages of *M. ciceri* USDA 3378 and *M. muleiense* CCBAU 83963. Our research found that USDA 3378 had a significant competitive advantage in soil, with a microbial share ranging from 84.6 to 100 % (Zhang et al., 2022). However, it remains unclear why *M. ciceri* USDA 3378 has a competitive nodulation advantage over *M. muleiense* CCBAU 83963. Subsequently, in the analysis of differential expression of competitive nodulation-related genes in *M. ciceri* USDA 3378 under symbiotic and non-symbiotic conditions, we found that the expression of the flagellar system-related gene *motA* changed significantly.

Flagella play critical roles in the establishment and progression of root nodule formation. Flagella are supramolecular motility structures composed of a basal body embedded in the cell wall and membrane, a proximal hook, and a distal filament. The basal body acts as a bidirectional rotary motor, generating the force across the cytoplasmic membrane. The hook and filament control the direction and propulsion of movement, respectively, (Minamino and Imada, 2015). Generally, rhizobia are motile bacteria with active flagella, capable of swimming and tumbling. This motility refers to their ability to move from the inoculation site on the seed surface to the plant root cells near the root tip, which they can invade. It also includes following the site of invasion as the root grows (El-Shemy, 2011). Flagellum-mediated chemotaxis is an essential determinant for rhizobial colonization and subsequent nodulation (Liu et al., 2018). Most bacteria that swim are propelled by flagellar filaments, which are driven by a rotary motor embedded in the cell wall and the cytoplasmic membrane. The motor is powered by proton flux (or in some species, sodium ion flux) (Mitsui and Ohshima, 2008). The operation of the motor is achieved through the highly coordinated interaction between a rotor and multiple stator units. The MotA/B stator complex, formed by the transmembrane proteins MotA and MotB, functions as a transmembrane proton channel, transferring protons and generating the driving force to the rotor. As a key component of the proton channel, MotA plays a critical role in the regulation of flagellar motility (Chang et al., 2021). In *Escherichia coli*, the *motA* gene is essential for flagellar rotation (Blair and Berg, 1990). In *Salmonella typhimurium*, the MotA protein is associated with flagellar rotation (Garza et al., 1995).

In this study, the *motA* gene was targeted for deletion in *M. ciceri* USDA 3378 strain. Using homologous recombination and biparental conjugation methods, a *motA* gene deletion mutant (Δ*motA*-3378) was successfully constructed. By comparing its physiological characteristics with the wild type, we analyzed the effects of *motA* gene deletion on the growth, motility, exopolysaccharide synthesis, biofilm formation, and competitive nodulation ability of *M. ciceri* USDA 3378. Additionally, this study aimed to explore how the *motA* gene contributes to the strong competitiveness of *M. ciceri* USDA 3378.

## Results

### *motA* deletion does not impair *M. ciceri* growth

The upstream and downstream homologous arms of the *motA* gene were amplified from the USDA 3378 genome using primers motA-up-F/R (KpnI, NdeI) and motA-down-F/R (ApaI, SacI). The upstream arm measured 538 bp and the downstream arm 551 bp (Supplementary Figure 1A). These homologous arms were seamlessly cloned, purified, and sequentially ligated into the plasmid pCM351 to create the recombinant plasmid pCM351::*motA* up-down, which was then transformed into *E. coli* DH5α. Using colony PCR to confirm the presence of the inserts, fragments of 538 bp (Supplementary Figure 1B) and 551 bp (Supplementary Figure 1C) were successfully amplified. Sequencing of the entire plasmid confirmed the expected construction of pCM351::*motA* up-down. This plasmid was transformed into *E. coli* S17-1, followed by conjugation with USDA 3378. Verification of the mutant was performed by colony PCR with primer *motA*-F/R, showing a 2,442 bp band for wild-type USDA 3378 (WT-3378) and a 2,288 bp band for the mutant strain (Δ*motA*-3378) (Supplementary Figure 1D). Sequencing of the mutants confirmed these results. And we produced a *motA* complementation strain of Δ*motA*-3378, Δ*motA*-3378-C (Supplementary Figures 2A–C).

**Figure 1:**
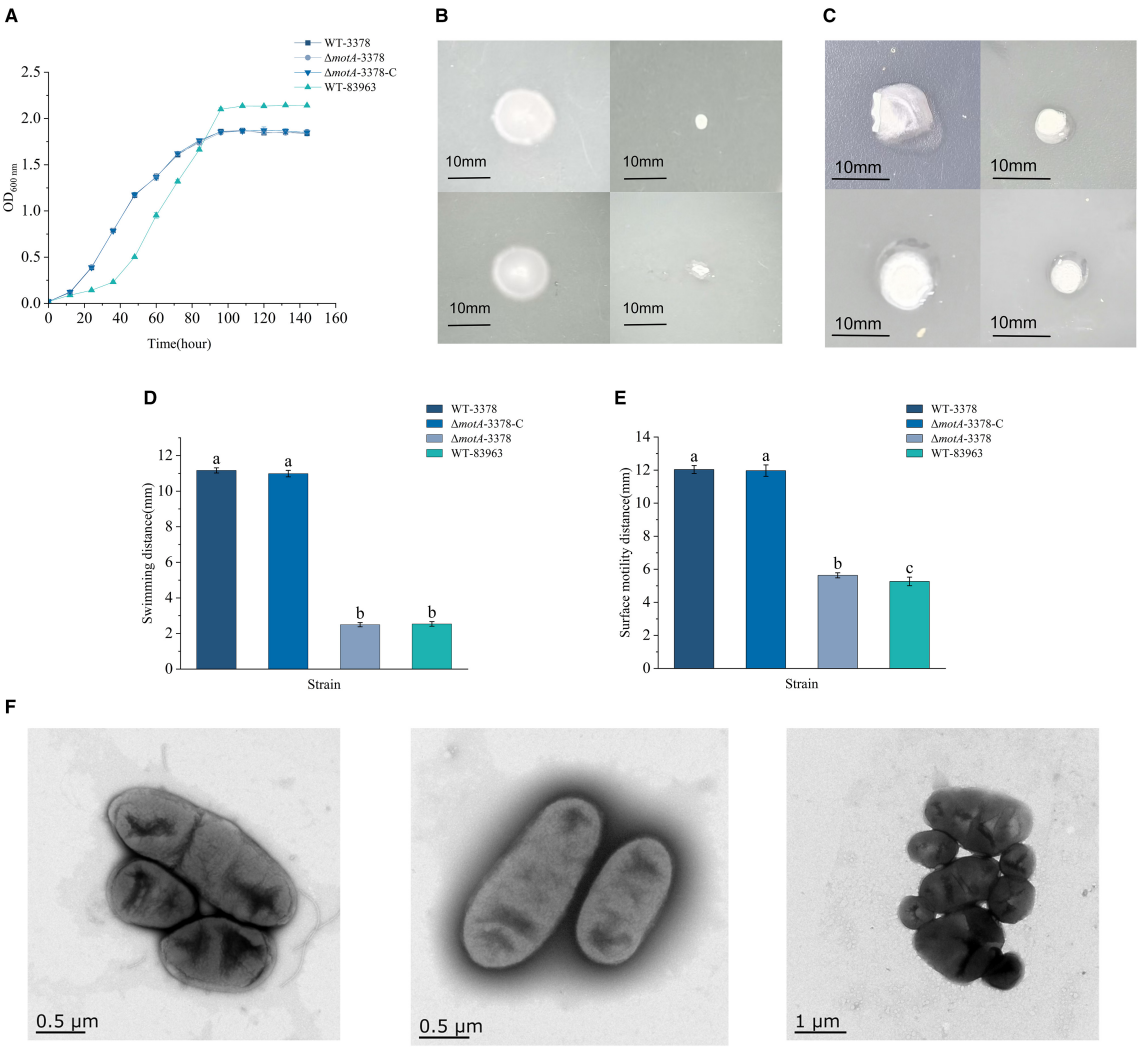
Motility, flagella production and growth curves rhizobial strains *M. ciceri* USDA 3378 (WT-3378), *M. ciceri* 3378 deficient in *motA* (Δ*motA*-3378), *M. ciceri* Δ*motA*-3378 complementation strain (Δ*motA*-3378-C) and *M. muleiense* CCBAU 83963T (WT-83963). Data are given as average (*n* = 3) ± standard deviation. Different letters indicate significant differences between treatments (*P* < 0.05) by one-way analysis of variance. Growth rates of WT-3378, Δ*motA*-3378 and Δ*motA*-3378-C were similar **(A)**. Under standard culture conditions, motility of WT-3378 and Δ*motA*-3378-C was similar and substantially greater than that of Δ*motA*-3378 or WT-83963 **(B, C, D, E)**. WT-3378 produced flagella but Δ*motA*-3378 and WT-83963 did not **(F)**.

After 144 h of incubation at 28 °C, no difference was observed in the growth ability between Δ*motA*-3378 and WT-3378, indicating that the deletion of *motA* did not affect the growth ability of *M. ciceri* (Figure 1A).

### *motA* gene mutation impairs flagella synthesis and motility in *M. ciceri*

Flagellum-based motility is known to enhance the ability of rhizobia to establish symbiosis with legume hosts. We assessed this in the Δ*motA*-3378. Swimming motility assays on 0.3 % agar-BM medium plates showed that WT-3378 exhibited significantly greater movement (11.16 ± 0.15 mm) compared to the Δ*motA*-3378 mutant (2.57 ± 0.12 mm) and WT-83963 (2.50 ± 0.12 mm) after 5 days of culture (Figures 1B, D). Similarly, surface motility on 0.4 % agarose MM medium plates revealed that WT-3378 traversed 12.03 ± 0.24 mm, while Δ*motA*-3378 and WT-83963 covered substantially shorter distances of 5.63 ± 0.15 mm and 5.27 ± 0.26 mm, respectively (Figures 1C, E). Importantly, complementation of the *motA* deletion in Δ*motA*-3378-C restored both swimming and surface motility to WT-3378 levels (Figures 1B–E). These results indicate that WT-3378 possesses superior motor ability compared to WT-83963, and that the *motA* gene is essential for robust motility in *M. ciceri*.

To correlate these motility observations with flagellar presence, the impact of the *motA* gene mutation on flagella in WT-3378 and the existence of flagella in strain WT-83963 were examined using transmission electron microscopy (TEM). TEM images clearly showed that WT-3378 possessed distinct, slender flagellar structures. Conversely, both the Δ*motA*-3378 mutant and the WT-83963 strain were entirely devoid of visible flagella (Figure 1F).

### Biofilm formation and extracellular polysaccharide synthesis were decreased in the Δ*motA*-3378 mutant

**Figure 2:**
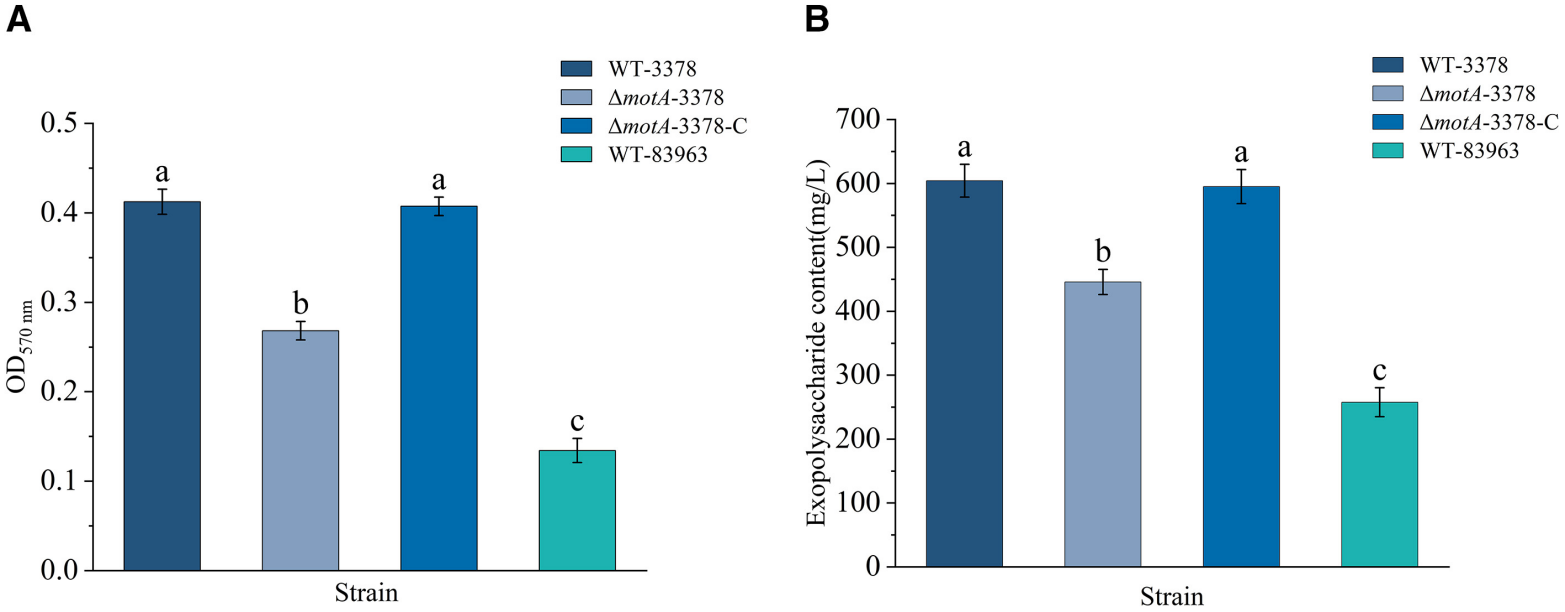
Biofilm and extracellular polysaccharide production of rhizobial strains *M. ciceri* USDA 3378^T^ (WT-3378), *M. ciceri* 3378 deficient in *motA* (Δ*motA*-3378), *M. ciceri* Δ*motA*-3378 complementation strain (Δ*motA*-3378-C) and *M. muleiense* CCBAU 83963^T^ (WT-83963). Data are given as average (*n* = 3) ± standard deviation. Different letters indicate significant differences between treatments (*P* < 0.05) by one-way analysis of variance. Biofilm production by WT-3378 and Δ*motA*-3378-C was similar and greater than that for Δ*motA*-3378 or WT-83963 **(A**). Extracellular polysaccharide production decreased with rhizobial strain in the order WT-3378≈Δ*motA*-3378-C >Δ*motA*-3378 > WT-83963 **(B**).

Biofilm production by the rhizobial strains was observed to decrease in the order of WT-3378 (OD_570nm_ = 0.412 ± 0.01), followed by Δ*motA*-3378 (OD_570nm_ = 0.268 ± 0.01), and then WT-83963 (OD_570nm_ = 0.134 ± 0.01) (Figure 2A). The OD_490nm_ values for various concentrations of glucose standard solutions were measured using the phenol-sulfuric acid method, and a standard curve was constructed (Supplementary Figure 3). The equation for the standard curve is y = 0.0081x + 0.025, *R*^2^ = 0.9938. A similar trend was observed for extracellular polysaccharide (EPS) production, with WT-3378 exhibiting the highest levels (604.27 ± 25.59 mg/L), which were significantly greater than those of Δ*motA*-3378 (445.83 ± 19.70 mg/L), and subsequently WT-83963 (257.73 ± 22.63 mg/L) (Figure 2B). Notably, both biofilm and EPS production levels for the complemented strain Δ*motA*-3378-C were comparable to those of the WT-3378 (Figures 2A, B). These results collectively indicate that the deletion of *motA* in *M. ciceri* significantly diminishes its capacity for both biofilm and extracellular polysaccharide production. Furthermore, WT-3378 consistently demonstrated superior production of both factors compared to WT-83963.

### Competitive nodulation ability of *M. ciceri* decreased after the deletion of *motA*

**Figure 3:**
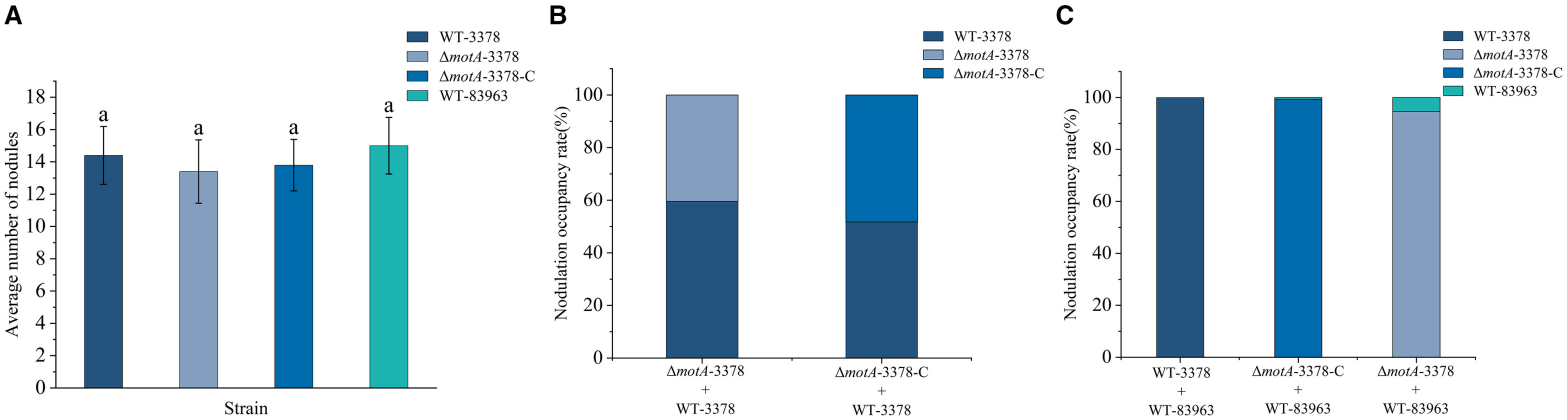
Nodule number of chickpea when inoculated individually and % nodule occupancy when inoculated with selected pairings of rhizobial strains *Mesorhizobium ciceri* USDA 3378 (WT-3378), *M. ciceri* 3378 deficient in *motA* (Δ*motA*-3378), *M. ciceri* Δ*motA*-3378 complementation strain (Δ*motA*-3378-C) and *M. muleiense* CCBAU 83963 (WT-83963) with or without additional extracellular polysaccharide. The data of A are given as average (*n* = 8) ± standard deviation, the same letter for each data point indicates that there was no significant difference between treatments (*P* > 0.05) on one-way analysis of variance. Nodule number was similar for all strains when inoculated individually **(A)**; Co-inoculation of Δ*motA*-3378 with WT-3378 resulted in a nodulation rate of 40.43% **(B)**; The nodulation rate of WT-3378 was 100% when mixed with WT-83963 but the nodulation rate for Δ*motA-*3378 was 94.60% when mixed with WT-83963 **(C)**.

After 30 days, nodules from chickpea plants subjected to various inoculation treatments were collected (Supplementary Figure 4A). No significant differences in root nodule numbers were observed among chickpea plants inoculated with different bacterial strains (Figure 3A). Nodule occupancy by the different strains was subsequently determined using PCR (Supplementary Figure 4B) or BOX-PCR (Supplementary Figure 4C). When Δ*motA-*3378 and WT-3378 were co-inoculated, the nodule occupancy of Δ*motA*-3378 was 40.43 % (Figure 3B), while WT-3378 occupied 59.57 % of the nodules. In competition with WT-83963, WT-3378 achieved 100 % nodule occupancy. In a separate competition experiment against WT-83963, Δ*motA*-3378 showed 94.6 % nodule occupancy (Figure 3C). These results indicate a reduction in the competitive nodulation ability of *M. ciceri* following the deletion of the *motA* gene.

## Discussion

In newly established planting areas in China, the introduced rhizobial inoculum *M. ciceri* USDA 3378 exhibits a competitive advantage over the indigenous *M. muleiense* CCBAU 83963 in nodulating chickpea (Zhang et al., 2022). To investigate the reasons behind the significant competitive nodulation advantage of *M. ciceri* USDA 3378 over *M. muleiense* CCBAU 83963, comparative genomic analysis of both strains, along with transcriptomic analysis of USDA 3378, revealed a notable change in the expression of the flagellar motor gene *motA* under symbiotic conditions.

Flagella play a crucial role in the survival and development of bacteria. Similar to other structures, the formation and expression of flagella are controlled by genes. Among these, proteins encoded by the *motA, motB, fliG, fliM*, and *fliN* genes are responsible for driving the flagellar motor, playing a key role in flagellar movement (Kenrick et al., 2011). The stator protein MotA can interact with the movement of the C-terminal domain of FliG (FliGC), thereby altering the rotor-stator interface to switch the direction of rotary motion (Terashima et al., 2008). MotA can also interact with MotI (motility inhibitor), separating it from the drivetrain protein, functioning like a molecular clutch to inhibit flagellar rotation (Subramanian et al., 2017).

In this study, the *motA* gene deletion mutant (Δ*motA*-3378) was successfully constructed from the WT-3378 using homologous recombination and bi-parental conjugation methods. To investigate whether the deletion of the *motA* gene affects the competitive nodulation ability of USDA 3378, we compared the growth characteristics and symbiotic properties of Δ*motA*-3378, WT-3378, and WT-83963. Deletion of the *motA* gene did not significantly alter the growth rate of *M. ciceri* USDA 3378 but markedly impaired its motility—a phenotype consistent with observations in *Campylobacter jejuni motA* mutants (Ruan et al., 2010). These findings collectively demonstrate the essential role of the flagellar motor gene *motA* in governing motility in *M. ciceri* USDA 3378. Transmission electron microscopy (TEM) confirmed the absence of flagellar filaments on Δ*motA*-3378 cells, explaining its motility deficiency. Additionally, the biofilm formation and extracellular polysaccharide production of the Δ*motA*-3378 mutant were significantly lower than those of USDA 3378, with reductions of 34.95 % and 26.20 %, respectively, indicating that the *motA* gene plays a role in the biofilm and extracellular polysaccharide formation capabilities of *M. ciceri*. Current consensus posits that biofilm development encompasses three sequential stages: initial attachment, maturation, and dispersal (Wang et al., 2020). Biofilm formation typically initiates when free-living bacteria transition to a surface-adherent state (Rumbaugh and Sauer, 2020). Given that the Δ*motA*-3378 mutant exhibits impaired motility, it fails to efficiently migrate to surfaces, thereby compromising the attachment phase and ultimately disrupting biofilm development. Since EPS constitute the primary matrix component of biofilms, diminished biofilm formation is concomitant with reduced EPS production. Furthermore, previous studies have shown that EPS can influence bacterial motility (Liu et al., 2016), mutants with impaired EPS biosynthesis exhibit diminished swimming motility (Nagórska et al., 2010). Flagellar rotation propels bacterial movement—an energy-intensive process that consumes substantial ATP to generate mucoid layers functioning as surfactants or wetting agents, thereby reducing surface tension between bacteria to facilitate propulsion (Kearns, 2010). In the flagellum-deficient Δ*motA*-3378 mutant, impaired motility eliminates the need for copious EPS production previously required to support coordinated bacterial locomotion. These mechanisms may collectively account for the significantly reduced biofilm formation and EPS production observed in the Δ*motA*-3378 compared to WT-3378, as documented earlier.

The competitive nodulation abilities of WT-3378, Δ*motA*-3378, and WT-83963 were tested on chickpeas over a period of 30 days. When mixed with WT-83963 as the inoculum, the nodulation rate of WT-3378 was 100 %, whereas the nodulation rate of Δ*motA*-3378 when mixed with WT-83963 was 94.6 % (Figure 3C). Additionally, the *motA* gene complemented strain Δ*motA*-3378-C exhibited a nodulation rate similar to that of WT-3378 when competing against WT-83963 (Figure 3C). Therefore, there is strong evidence to suggest that the *motA* gene plays a role in the enhanced competitive nodulation ability of *M. ciceri* against *M. muleiense*, potentially through its involvement in flagella production and cellular motility, as well as its effects on biofilm and EPS production. Previous studies have demonstrated that motility plays an important role in the colonization of plant hosts by various rhizobial species (Tambalo et al., 2015). Surface polysaccharides of rhizobia—including EPS—function as signaling molecules and counteract plant defense responses during symbiotic interactions, thereby facilitating successful nodulation (Vinardell et al., 2004; Janczarek et al., 2015). However, the Δ*motA*-3378 mutant is still with a competitive nodulation rate of 94.6 %, indicating that it retains a strong competitive nodulation ability compared to *M. muleiense*. This suggests that the *motA* gene can affect the competitive nodulation ability of *M. ciceri* USDA 3378 in multiple ways, but it is not the sole factor contributing to the stronger competitive nodulation ability of *M. ciceri* against *M. muleiense*.

In summary, this study investigated the impact of the flagellar motor gene *motA* on physiological traits of *M. ciceri* USDA 3378, contributing new insights into the regulatory role of flagellar motors in rhizobial symbiosis. Critically, the presence of *motA* confers enhanced competitive nodulation capacity to *M. ciceri* in chickpea. However, *motA* is not the sole determinant underlying the superior competitive nodulation of *M. ciceri* relative to *M. muleiense* CCBAU 83963, necessitating further investigations to fully elucidate the mechanistic distinctions between these strains.

## Materials and methods

### Bacterial strains, plasmids, and growth conditions

The strains and plasmids used in this study are detailed in Table 1. The *Escherichia coli* strain was cultured in Luria-Bertani (LB) medium at 37 °C (Denman, 1983), with or without 1.8 % agar, supplemented with appropriate antibiotics: kanamycin (50 μg/mL) and gentamicin (30 μg/mL). *M. ciceri* USDA 3378 and *M. muleiense* CCBAU 83963 were grown in Tryptone-Yeast (TY) medium (Breinger, 1974) and Modified-Yeast-Mannitol Agar (M-YMA) medium (Zhang et al., 2012) at 28 °C, with or without 1.8 % agar. The plasmid was introduced into *E. coli* via heat shock at 42 °C and into *M. ciceri* USDA 3378 through biparental mating (Figureurski and Helinski, 1979; Thoma and Schobert, 2009).

**Table 1:** Bacterial strains, plasmids, and seeds used in this study.

**Materials**	**Characteristics**	**Reference or source**
**Strains**		
*M. ciceri* USDA 3378	The host is the chickpea	
	Isolated from Japan; Kan^r^	Lab stock
*M. muleiense* CCBAU 83963	The host is the chickpea	
	Isolated from Xinjiang, China; Chl^r^	Lab stock
Δ*motA-*3378	*motA* mutant strain based on USDA 3378; Kanr, Gm^r^	This study
*E. coli* DH5α	lac ZΔM15, recA gyrA	Purchased from TaKaRa
*E. coli* S17-1	recA pro (RP4-2Tet::Mu Kan::Tn7)	From Huazhong Agricultural University
**Plasmids**		
pCM351	Cre-loxP recombinant system plasmid vector; Gm^r^	From Huazhong Agricultural University
pCM351::*motA* up-down	pCM351 with upstream and downstream homologous arms of *motA*; Gm^r^	This study
pCM157	Cre recombinase Bacterial Expression; Tet^r^	From Huazhong Agricultural University
pBBR1MCS-5	Broad-spectrum host plasmid; Gm^r^	From Huazhong Agricultural University
pBBR1MCS-5-*motA*	PBBR1MCS-5 connects the complementary sequence of *motA*; Gm^r^	This study
**Seeds**		
Chickpea	The Kabuli variety, from Xinjiang, China	Lab stock

### Whole genome sequencing

The whole genomes of USDA 3378 and CCBAU 83963 were sequenced and submitted to the NCBI database. The genomic sequences for these two rhizobial chickpea strains are available on NCBI, with accession numbers NZ_FNEE01000000.1 for WT-3378 and NZ_JAKHFU010000000.1 for WT-83963 (= CGMCC 1.11022). By comparing the distribution and number of functional genes in the genomes of chickpea rhizobial strains USDA 3378 and CCBAU 83963, systematic gene clusters were analyzed using the Genelibs online platform (https://www.genelibs.com/gb/pages/login.jsf). Further analysis focused on differences in genes related to competitive nodulation from a molecular biology perspective.

### Transcriptome sequencing under symbiotic and non-symbiotic conditions

Following established methods (Liu et al., 2018; Zamudio and Bastarrachea, 1994; Zhang et al., 2022), selected chickpea seeds were placed in a sterilized conical flask, sterilized with 95 % ethanol, and then surface-sterilized by rinsing with 2.5 % (*v/v*) NaClO for 4 mins. They were rinsed seven times with sterile water and cultured in the dark at 28 °C for 4 days. Healthy chickpea sprouts without surface secretions were immersed in a USDA 3378 bacterial solution (OD_600nm_ = 1.0). Twelve sprouts were added to each flask and incubated at 28 °C and 50 rpm for 2 h to simulate symbiotic conditions. Additionally, a USDA 3378 culture (OD_600nm_ = 1.0) without sprouts was incubated under the same conditions to represent non-symbiotic conditions. Bacteria were collected by centrifugation at 12,000 rpm for 5 mins, and both treatments were repeated three times. The bacterial samples were stored at −80 °C and sent for commercial sequencing (Sangon Biotech, Shanghai Co., Ltd.).

### Construction of the *motA* mutant and its complemented strain

The upstream and downstream homologous arms of the *motA* gene were amplified using primers *motA*-up-F/R (KpnI, NdeI) and *motA*-down-F/R (ApaI, SacI) with the wild-type *M. ciceri* USDA 3378 genome as the template. These homologous arms and plasmid pCM351 were digested, purified, and ligated into pCM351 using the Hieff Clone Universal One Step Cloning Kit to create the recombinant plasmid pCM351::*motA*up-down. The entire plasmid was sequenced to confirm accuracy. The correctly sequenced plasmid was transformed into *E. coli* S17-1, followed by biparental mating with WT-3378. Double-crossover mutants were selected on TY agar plates containing kanamycin and gentamicin. PCR verification of mutants was performed using primers motA-F and motA-R.

Δ*motA*-3378 (containing the gentamicin resistance gene) was conjugated with *E. coli* S17-1 containing plasmid pCM157 (expressing Cre recombinase). The gentamicin resistance gene in Δ*motA*-3378 was then eliminated by screening on TY agar plates supplemented with kanamycin (30 μg/mL) and tetracycline (20 μg/mL) to select for cells that had lost the pCM157 plasmid. Primers *motA*-F and *motA*-R were used to verify the absence of the gentamicin resistance gene in Δ*motA*-3378. The complete *motA* gene sequence (including the 605 bp upstream of the start codon) was amplified using primers AH-F/R (ApaI, SacI) with the wild-type USDA 3378 genome as the template. The *motA* complementation fragment and plasmid pBBR1MCS-5 were purified, digested, and ligated using the Hieff Clone Universal One-Step Cloning Kit to generate the recombinant plasmid pBBR1MCS-5-*motA*. Colony PCR with universal primers M13F/R was performed to verify successful ligation, followed by sequencing. The correctly sequenced plasmid was transformed into *E. coli* S17-1, and the resultant strain was conjugated with the gentamicin-sensitive Δ*motA*-3378. Complemented strains of Δ*motA*-3378 were selected on TY agar plates supplemented with kanamycin (30 μg/mL) only (since the strain is gentamicin-sensitive and the plasmid confers kanamycin resistance). Finally, the complementary strains were verified by PCR and sequencing with M13F/R. All primers used in this study are listed in Table 2.

**Table 2:** Primers were used in construction of mutant and complementary strains of *motA* gene.

**Primer**	**Sequence (5^′^-3^′^)**	**Fragment size (bp)**	**Target gene**
motA*-*up-F	CTGAATTCAGCTGTAC AATTGGTACCGCCTGG ATGGCATAGTTCTT	538	*motA* upstream homology arm
motA*-*up-R	ATACGAAGTTATGCGG CCGCCATATGGACAGG TCCTTCCGCATTTC		
motA*-*down-F	ATCCAGCTTATCGATA CCGCGGGCCCACGAT GCGCGATAGATCGGT	551	*motA* downstream homology arm
motA*-*down-R	GGTCGGCTGGATCC TCTAGTGAGCTCATCGC GCAGCACATACCGCT		
motA-F	AGTCGAGACCGAATG ACGCCA	2288(Δ*motA*-3378)/ 2242(WT-3378)	Whole region of *motA* for identification
motA-R	TGATCGTCGTCGCTGA CAGGCA		
AH-F	ACTCACTATAGGGCGA ATTGGAGCTCGTTGGC AAGATAGTATGTCG	1481	Complementary sequence of *motA*
AH-R	GGGAACAAAAGCTGGG TACCGGGCCCTCAGG CGGCCTTCTTTTC		
M13F	CGCCAGGGTTTTCCCA GTCACGAC		
M13R	AGCGGATAACAATTTC ACACAGG		

### Construction of growth curve

To assess the impact of the *motA* mutation on the growth of *M. ciceri*, growth curves were determined for strains Δ*motA*-3378, WT-3378, and WT-83963. All strains were cultured and adjusted to the same density (OD_600nm_ = 1.0). The bacterial suspensions were then inoculated into fresh M-YMA liquid medium at a 1 % inoculation rate and incubated at 28 °C with shaking at 180 rpm. Growth was monitored by measuring OD600 nm every 12 h over a 144 h period. This extended incubation time was selected to ensure the observation of all growth phases (lag, log, and stationary), consistent with the intermediate growth rate of *Mesorhizobium* species (Jarvis et al., 1982, 1997).

### Motility test

In order to compare the difference in motility between Δ*motA*-3378, WT-3378 and WT-83963, semisolid culture medium was used to test the motility (Zheng et al., 2015; Fuentes-Romero et al., 2024). All three strains were inoculated into 5 mL of TY broth and cultured at 28 °C with shaking at 180 rpm until the optical density at 600 nm reached 1.0. An equal volume (3 μL) of each strain was aspirated and inoculated into 0.3 % agar BM medium. Plates were sealed with Parafilm, incubated upright at 28 °C for 24 h, then inverted and further incubated at 28 °C for 4 days. Finally, the swimming distance of the strains was measured to quantitatively analyze their swimming motility. Each test was performed in triplicate.

Additionally, a surface motility assay was performed. The three test bacterial strains were cultured in TY broth to an OD600 nm of 1.0, using the same method as for the swimming motility assay. 1 mL of bacterial culture was transferred to a sterile 1.5 mL microcentrifuge tube and centrifuged at 12,000 × g for 3 mins. The pellet was washed twice with 1 mL of liquid MM medium, the supernatant was discarded, and the pellet was gently resuspended in 100 μL of liquid MM medium. A 2 μL aliquot of the bacterial suspension was spotted onto the center of a 0.4 % agarose MM medium plate. After drying at room temperature for 10 mins, plates were sealed with Parafilm and incubated inverted at 28 °C for 5 days. After 120 h of incubation at 28 °C, plates were stored at 4 °C for 2 days before motility distance measurement. Each test was performed in triplicate.

### Transmission electron microscope (TEM)

The Δ*motA*-3378, WT-3378, and WT-83963 strains were examined using a transmission electron microscope (Tecnai 12, Philips, Netherlands), following the method (Loferer-Krossbacher et al., 1998). A drop of bacterial suspension was placed on a slide, and a copper grid was applied to the drop for 2 mins. The grid was then removed, allowed to sit at room temperature for 3 mins, and excess liquid was carefully removed with absorbent paper. A drop of 5 % phosphotungstic acid was used for negative staining on the grid. After 1 min, excess stain was removed, the grid was rinsed with deionized water, and dried overnight. The samples were subsequently observed under the transmission electron microscope (Xiong et al., 2024).

### Detection of biofilm formation capacity

The qualitative and quantitative determination of biofilm formation ability of rhizobia followed modified methods from previous literature (Zhang et al., 2022). The experimental steps were as follows: Δ*motA*-3378, WT-3378, and WT-83963 were inoculated into 5 mL of M-YMA liquid medium and cultured at 28 °C with shaking at 180 rpm, and the cultures were adjusted to the same cell density (OD_600nm_ = 1.0). Each culture was then inoculated into 1.5 mL of M-YMA liquid medium with a 10 % (*v/v*) inoculum. The cultures were incubated at 28 °C for 7 days. After incubation, the liquid was discarded, and the tubes were washed three times with deionized water. A 0.1 %(*w/v*) crystal violet solution (2 mL) was added to each tube for staining for 30 mins. The dye was then discarded, the tubes were washed three times with deionized water, and air-dried upside down to observe biofilm formation on the inner walls. For quantitative analysis, 3 mL of 30 %(*v/v*) acetic acid was added to each tube and left for 2 h. The OD_570nm_ was measured for each tube, with each test repeated at least three times.

### Determination of the ability to produce extracellular polysaccharide

A standard curve was generated using the phenol-sulfuric acid method, following (Yadav et al., 2024). The extracellular polysaccharides produced by Δ*motA*-3378, WT-3378, and WT-83963 were quantitatively determined using the same method. The procedure is as follows: Strains were cultured to the same density (OD_600nm_ = 1.0) and inoculated at 1 % (*v/v*) into M-YMA liquid medium. After reaching the same density, the cultures were centrifuged at 4 °C, 4,000 rpm for 15 mins. The supernatant was collected, mixed with four times its volume of 96 % (*v/v*) ethanol, and left at 4 °C for 30 mins to form white flocs. After centrifugation at 4 °C, 10,000 rpm for 15 mins, the supernatant was discarded, and the precipitate was dried overnight. Each experiment was performed in triplicate.

To prepare the extracellular polysaccharide solution, 5 mL of deionized water was added to dissolve the dried precipitate. This was followed by 20 fold dilution with deionized water, and 1 mL of the sample was mixed with 0.5 mL of 6 % (*w/v*) phenol solution and 5 mL of concentrated sulfuric acid, and allowed to stand for 20 mins. The reaction was compared to a blank control using deionized water. The OD_490nm_ values were measured, and the extracellular polysaccharide content was calculated using a glucose standard curve.

### Determination of competitive nodulation ability

To determine the competitive nodulation ability of Δ*motA*-3378, chickpea seeds were co-inoculated with Δ*motA*-3378 and either WT-3378 or WT-83963 in a pot experiment. The strains (Δ*motA*-3378, WT-3378, and WT-83963) were prepared by culturing them in M-YMA liquid medium and adjusted to the same density (OD_600nm_ = 1.0). Sterilized chickpea seeds (soaked in 2.5 % (v/v) sodium hypochlorite solution for 4 mins) were germinated and sown in sterilized plastic pots (15 cm × 10 cm) filled with a sterilized vermiculite mixture (Garrity et al., 2005). Two mixed inoculation treatments were applied to the chickpea sprouts: (1) 1 mL Δ*motA*-3378 and 1 mL WT-3378, (2) 1 mL Δ*motA*-3378 and 1 mL WT-83963, (3) 1 mL WT-3378 and 1 mL WT-83963. Three single inoculation treatments served as controls: 1 mL Δ*motA*-3378, 1 mL WT-3378, and 1 mL WT-83963. Each treatment had 10 replicates, and plants were grown in a greenhouse at 25/20 °C (day/night) with a 16 h light period. After 30 days, nodules were harvested and disinfected (soaked in 2.5 % (*v/v*) sodium hypochlorite solution for 4 mins). To assess competitive nodulation, the identity of the occupying strain within each nodule was determined. In competition experiments between Δ*motA*-3378 and WT-3378, PCR amplification was performed on DNA templates derived from nodule extracts using specific primers, motA-F and motA-R, to distinguish between the two strains. For competitive nodulation assays involving WT-3378 or Δ*motA*-3378 against WT-83963, nodule extracts were used as DNA templates for BOX-PCR amplification with the single primer BOX-A1R, enabling the identification of the specific strain that formed each nodule.

### Statistical analysis

Statistical analysis was performed with SPSS v.21.0 and Origin 2024 software. Data are presented as the mean ± standard deviation (SD). Each experiment was repeated at least three times. Differences were considered significant when *P* < 0.05.

## Data Availability

The datasets presented in this study can be found in online repositories. The names of the repository/repositories and accession number(s) can be found in the article/Supplementary material.
