# Supplementary material for: motA-mediated flagellar motility modulates biofilm formation and competitive nodulation in Mesorhizobium ciceri USDA 3378
Source: Front Microbiol. 2026 Jan 21;16:1743961. doi: 10.3389/fmicb.2025.1743961 (PMC12869990; doi:10.3389/fmicb.2025.1743961)
Supplement: Supplementary file 1 [file Presentation_1.pptx]

## Slide 1
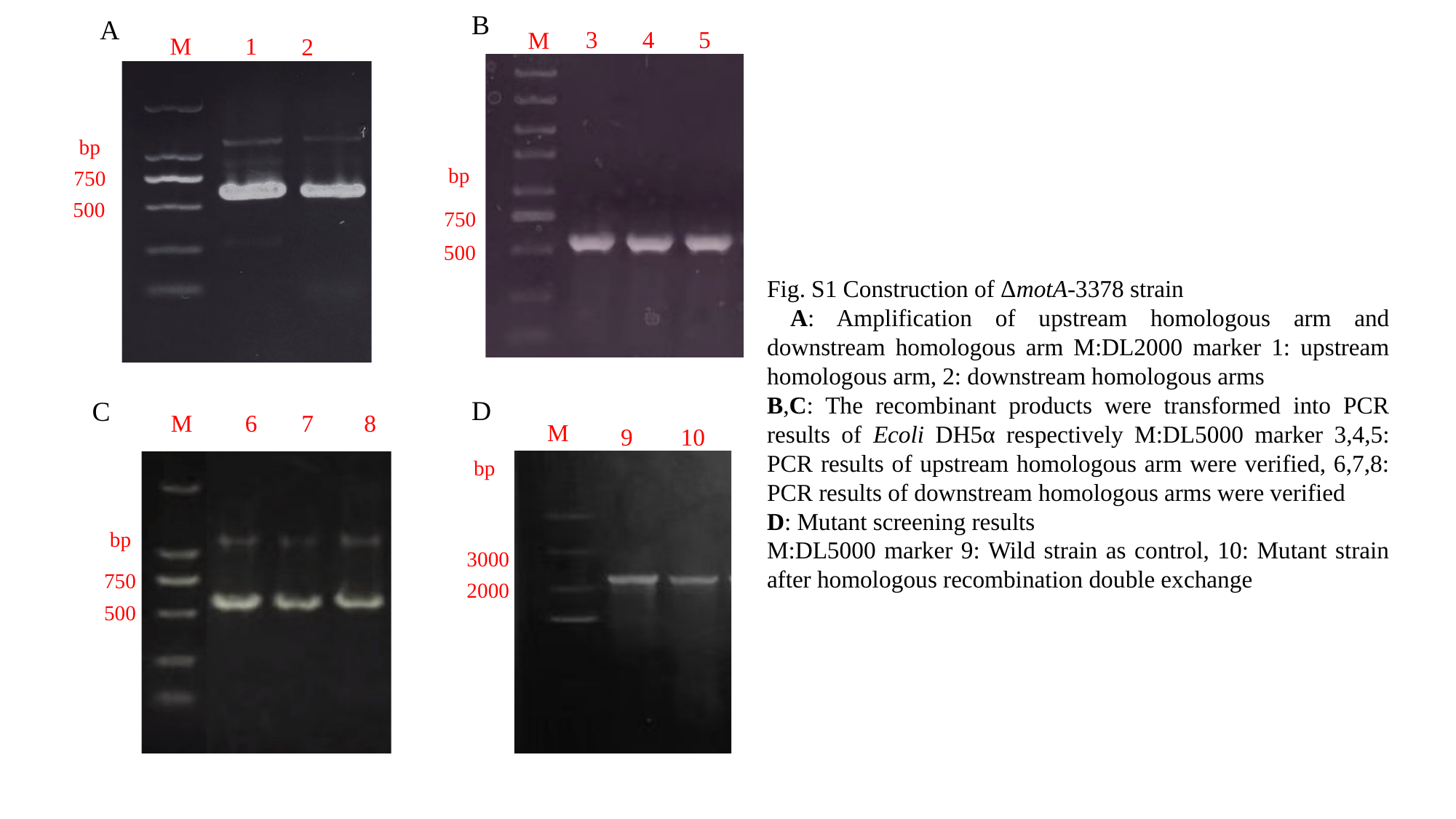

B
A
3
4
5
M
M
1
2
bp
bp
750
500
750
500
Fig. S1 Construction of ΔmotA-3378 strain
 A: Amplification of upstream homologous arm and downstream homologous arm M:DL2000 marker 1: upstream homologous arm, 2: downstream homologous arms
B,C: The recombinant products were transformed into PCR results of Ecoli DH5α respectively M:DL5000 marker 3,4,5: PCR results of upstream homologous arm were verified, 6,7,8: PCR results of downstream homologous arms were verified
D: Mutant screening results
M:DL5000 marker 9: Wild strain as control, 10: Mutant strain after homologous recombination double exchange
D
C
8
M
6
7
M
9
10
bp
bp
3000
750
2000
500

## Slide 2
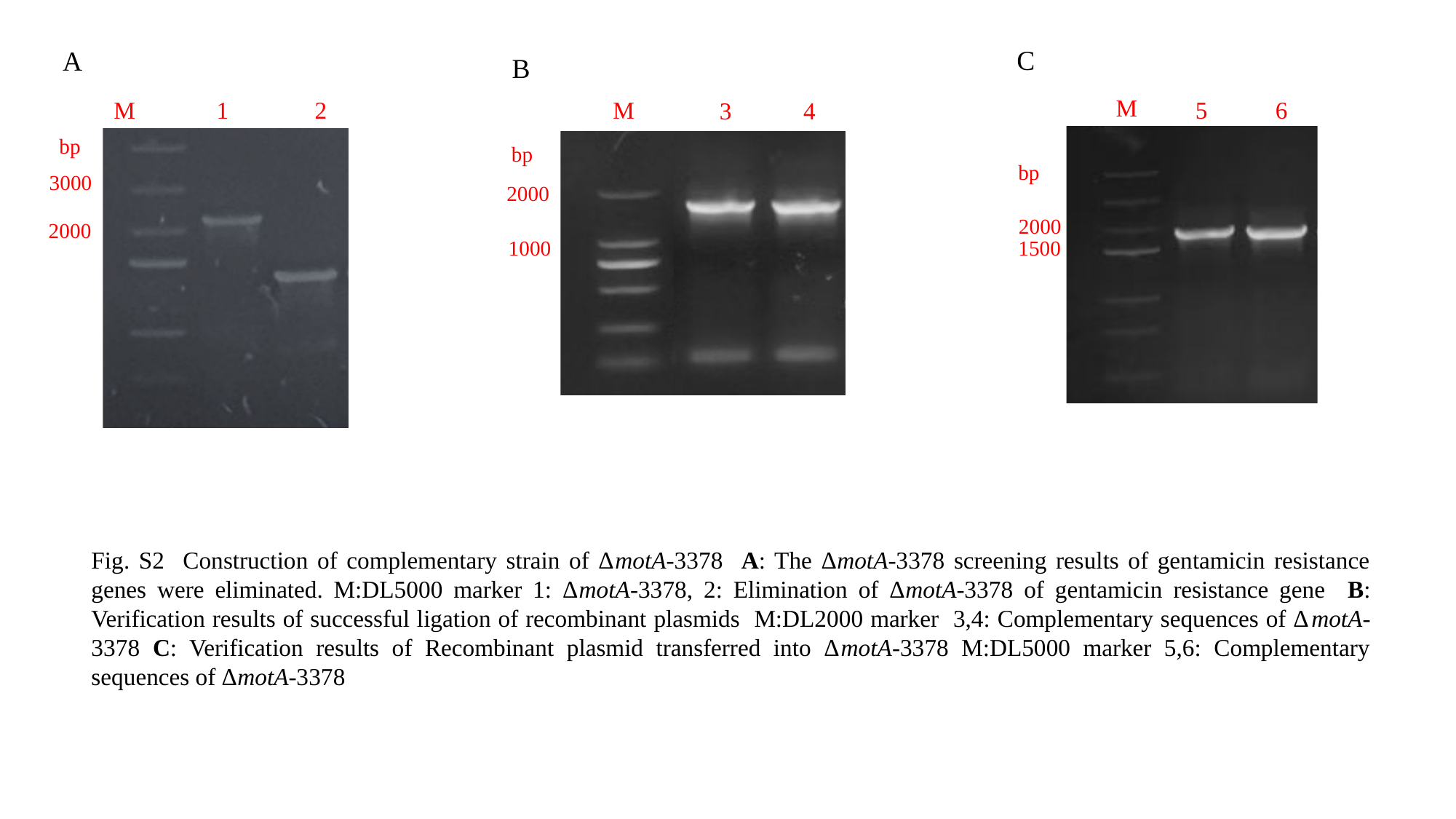

C
A
B
M
M
1
2
M
5
6
3
4
bp
bp
bp
3000
2000
2000
2000
1500
1000
Fig. S2 Construction of complementary strain of ΔmotA-3378 A: The ΔmotA-3378 screening results of gentamicin resistance genes were eliminated. M:DL5000 marker 1: ΔmotA-3378, 2: Elimination of ΔmotA-3378 of gentamicin resistance gene B: Verification results of successful ligation of recombinant plasmids M:DL2000 marker 3,4: Complementary sequences of ΔmotA-3378 C: Verification results of Recombinant plasmid transferred into ΔmotA-3378 M:DL5000 marker 5,6: Complementary sequences of ΔmotA-3378

## Slide 3
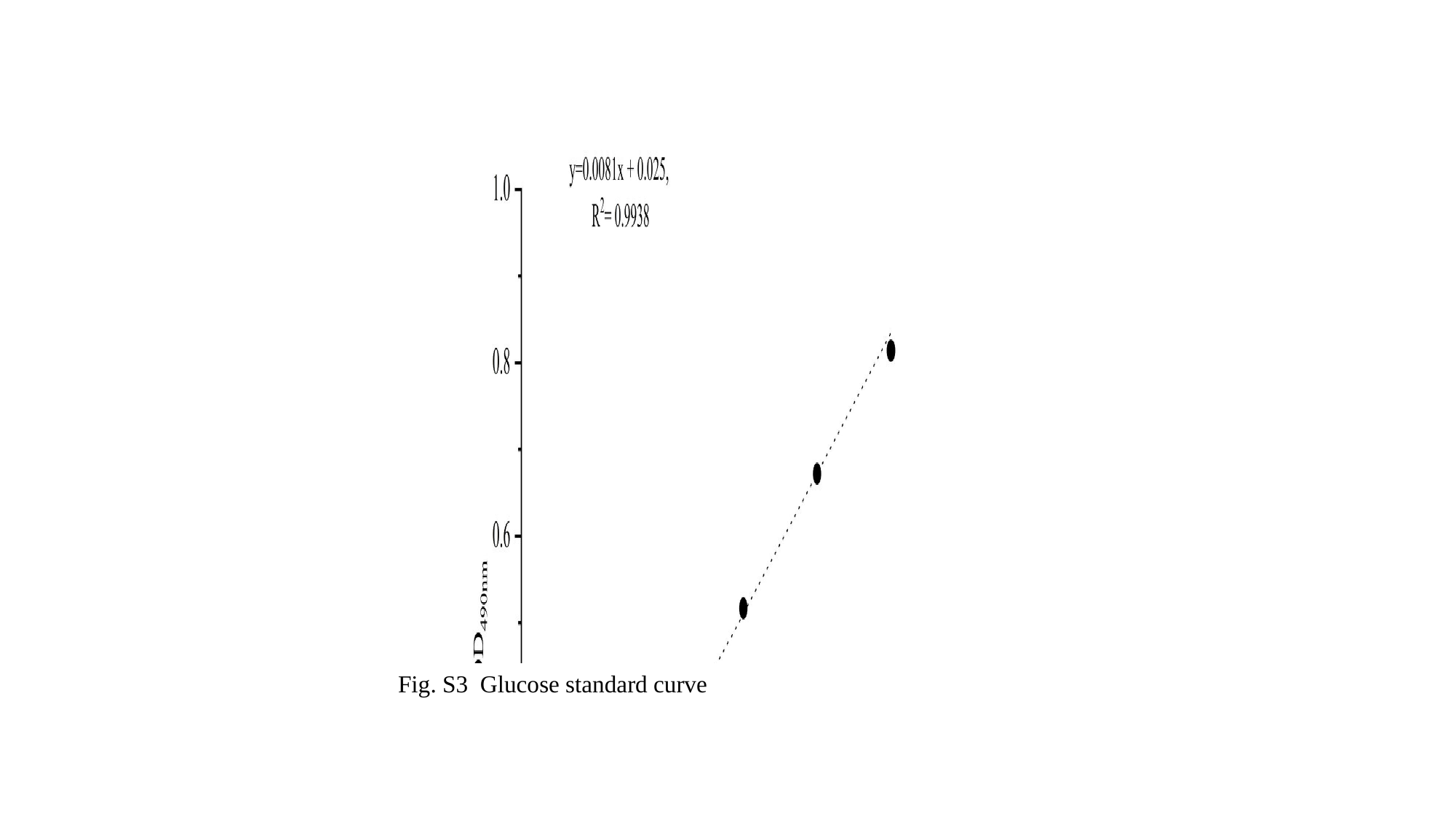

Fig. S3 Glucose standard curve

## Slide 4
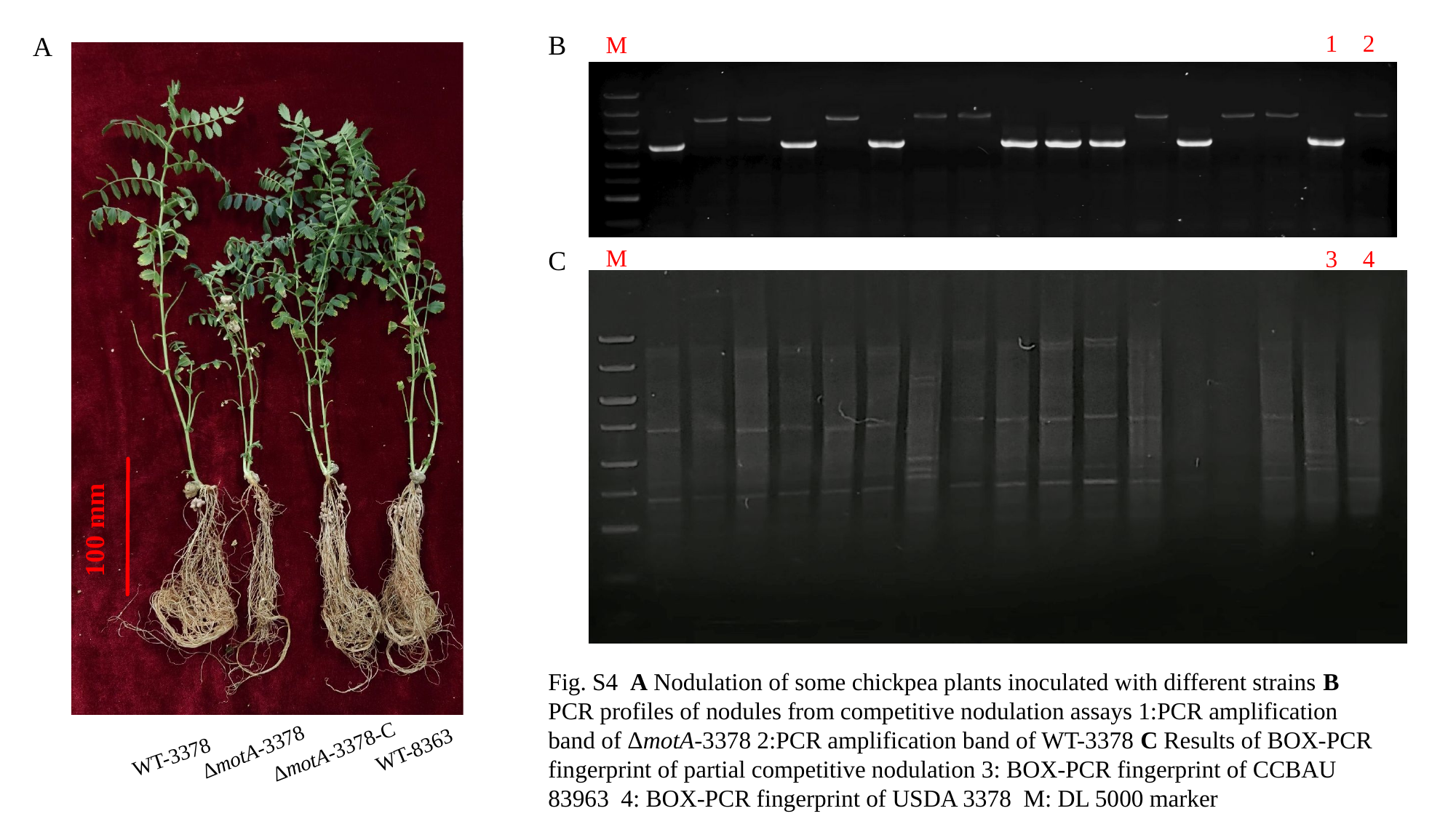

1
1
2
B
M
A
M
3
4
C
100 mm
Fig. S4 A Nodulation of some chickpea plants inoculated with different strains B PCR profiles of nodules from competitive nodulation assays 1:PCR amplification band of ΔmotA-3378 2:PCR amplification band of WT-3378 C Results of BOX-PCR fingerprint of partial competitive nodulation 3: BOX-PCR fingerprint of CCBAU 83963 4: BOX-PCR fingerprint of USDA 3378 M: DL 5000 marker
WT-8363
ΔmotA-3378
ΔmotA-3378-C
WT-3378
